# Comparative incidence of early and late bloodstream and respiratory tract co-infection in patients admitted to ICU with COVID-19 pneumonia versus Influenza A or B pneumonia versus no viral pneumonia: wales multicentre ICU cohort study

**DOI:** 10.1186/s13054-022-04026-9

**Published:** 2022-06-02

**Authors:** Manish Pandey, Alexander May, Laura Tan, Harriet Hughes, Jack Parry Jones, Wendy Harrison, Scott Bradburn, Sam Tyrrel, Babu Muthuswamy, Nidhika Berry, Richard Pugh, Daryn Sutton, Andy Campbell, Matthew Morgan

**Affiliations:** 1 Adult Critical Care Directorate, University Hospital of Wales, Heath Park, Cardiff, UK; 2 Department of Microbiology and Infectious Diseases, University Hospital of Wales, Heath Park, Cardiff, UK; 3 Senior Scientist, Healthcare Associated Infection, Antimicrobial Resistance & Prescribing Programme (HARP), Public Health Wales, Cardiff, UK; 4 Adult Critical Care Directorate, Swansea Bay Health Board, Swansea, UK; 5 Department of Critical Care, Grange University Hospital, Cwmbran, UK; 6 Department of Clinical Microbiology and Infection Control, Royal Gwent Hospital, Newport, UK; 7 Department of Anaesthesia, Glan Clwyd Hospital, Bodelwyddan, UK; 8 Advanced Epidemiological Scientist, Healthcare Associated Infection, Antimicrobial Resistance & Prescribing Programme (HARP), Public Health Wales, Cardiff, UK; 9 Adult Critical Care Directorate, Wrexham Maelor Hospital, Wrexham, UK

**Keywords:** COVID-19, Influenza A and B, SARS-CoV-2, Co-infection, Early, Late, Antibiotic sensitivity, Aspergillus

## Abstract

**Objective:**

The aim is to characterise early and late respiratory and bloodstream co-infection in patients admitted to intensive care units (ICUs) with SARS-CoV-2-related acute hypoxemic respiratory failure (AHRF) needing respiratory support in seven ICUs within Wales, during the first wave of the COVID-19 pandemic. We compare the rate of positivity of different secondary pathogens and their antimicrobial sensitivity in three different patient groups: patients admitted to ICU with COVID-19 pneumonia, Influenza A or B pneumonia, and patients without viral pneumonia.

**Design:**

Multicentre, retrospective, observational cohort study with rapid microbiology data from Public Health Wales, sharing of clinical and demographic data from seven participating ICUs.

**Setting:**

Seven Welsh ICUs participated between 10 March and 31 July 2020. Clinical and demographic data for COVID-19 disease were shared by each participating centres, and microbiology data were extracted from a data repository within Public Health Wales. Comparative data were taken from a cohort of patients without viral pneumonia admitted to ICU during the same period as the COVID-19 cohort (referred to as no viral pneumonia or ‘no viral’ group), and to a retrospective non-matched cohort of consecutive patients with Influenza A or B admitted to ICUs from 20 November 2017. The comparative data for Influenza pneumonia and no viral pneumonia were taken from one of the seven participating ICUs.

**Participants:**

A total of 299 consecutive patients admitted to ICUs with COVID-19 pneumonia were compared with 173 and 48 patients admitted with no viral pneumonia or Influenza A or B pneumonia, respectively.

**Main outcome measures:**

Primary outcome was to calculate comparative incidence of early and late co-infection in patients admitted to ICU with COVID-19, Influenza A or B pneumonia and no viral pneumonia. Secondary outcome was to calculate the individual group of early and late co-infection rate on a per-patient and per-sample basis, with their antimicrobial susceptibility and thirdly to ascertain any statistical correlation between clinical and demographic variables with rate of acquiring co-infection following ICU admission.

**Results:**

A total of 299 adults (median age 57, M/F 2:1) were included in the COVID-19 ICU cohort. The incidence of respiratory and bloodstream co-infection was 40.5% and 15.1%, respectively. *Staphylococcus aureus* was the predominant bacterial pathogen within the first 48 h. Gram-negative organisms from Enterobacterales group were predominantly seen after 48 h in COVID-19 cohort. Comparative no viral pneumonia cohort had lower rates of respiratory tract infection and bloodstream infection. The influenza cohort had similar rates respiratory tract infection and bloodstream infection. Mortality in all three groups was similar, and no clinical or demographic variables were found to increase the rate of co-infection and ICU mortality.

**Conclusions:**

Higher incidence of bacterial co-infection was found in COVID-19 cohort as compared to the no viral pneumonia cohort admitted to ICUs for respiratory support.

**Supplementary Information:**

The online version contains supplementary material available at 10.1186/s13054-022-04026-9.

## Introduction

Severe acute respiratory syndrome coronavirus-2 (SARS-CoV-2) is a novel beta coronavirus, first identified from a cluster of pneumonia cases in the city of Wuhan, China [1], in 2019. It subsequently spread rapidly across the globe including Wales, affecting millions of people from different ages, and resulting in an illness named coronavirus disease 2019 (COVID-19). As of 1 December 2021, the cumulative confirmed cases and deaths in Wales stand at 540,550 and 6212, respectively [2]. Damage to the respiratory epithelial lining, up-regulation and exposure of receptors, dampening of immune response or enhancement of inflammation and immune dysregulation are believed to facilitate establishment of bacterial or fungal co-infection in patients with initial viral insults [3]. Bacterial co-infection has been reported as the predominant cause of death rather than direct viral insult during previous viral pandemics [4, 5].

Historically, variable rates of co-infection with bacteria, fungi and other viruses have been described in patients admitted to healthcare settings, during endemic or pandemic viral illnesses caused by either Influenza, SARS (severe acute respiratory syndrome) or MERS (Middle East respiratory syndrome) viruses [6–14].

We aimed to identify the incidence of early (defined as growth of pathogen from blood culture or respiratory tract culture specimen within 48 h of hospital admission) and late (defined as growth of pathogen from blood culture or respiratory tract culture specimen after 48 h of hospital admission) co-infection in patients with COVID-19 pneumonia, Influenza A or B pneumonia or no viral pneumonia admitted to ICUs, requiring either basic or advanced respiratory support. Secondary aims were to calculate rates of bloodstream infection (BSI) and respiratory tract infection (RTI) and to characterise individual pathogens and report its antimicrobial sensitivities.

## Methods

### Study design

We conducted a retrospective, multicentre, observational, cohort study that enrolled patients from seven ICUs in Wales. Inclusion criterion were: (1) age more than or equal to 18 years, (2) admission to ICU for AHRF needing either basic [oxygen more than 50% through face mask or non-invasive ventilation (NIV) through a hood or mask] or advanced respiratory support [invasive mechanical ventilation (IMV)] and one of the following diagnostic criteria at ICU admission: (3A) pneumonia caused by SARS-CoV-2 viral infection identified by positive nasopharyngeal, throat swab or bronchoalveolar washings for SARS-CoV-2 virus, (3B) pneumonia caused by Influenza A or B identified by positive throat swab, respiratory secretions from bronchoalveolar washings, (3C) ICU admission for medical or surgical reasons with no precipitating viral pneumonia. Exclusion criteria were age less than 18 years and ICU admission with any other respiratory viral infection other than SARS-CoV-2 and Influenza A or B.

**Fig. 1:**
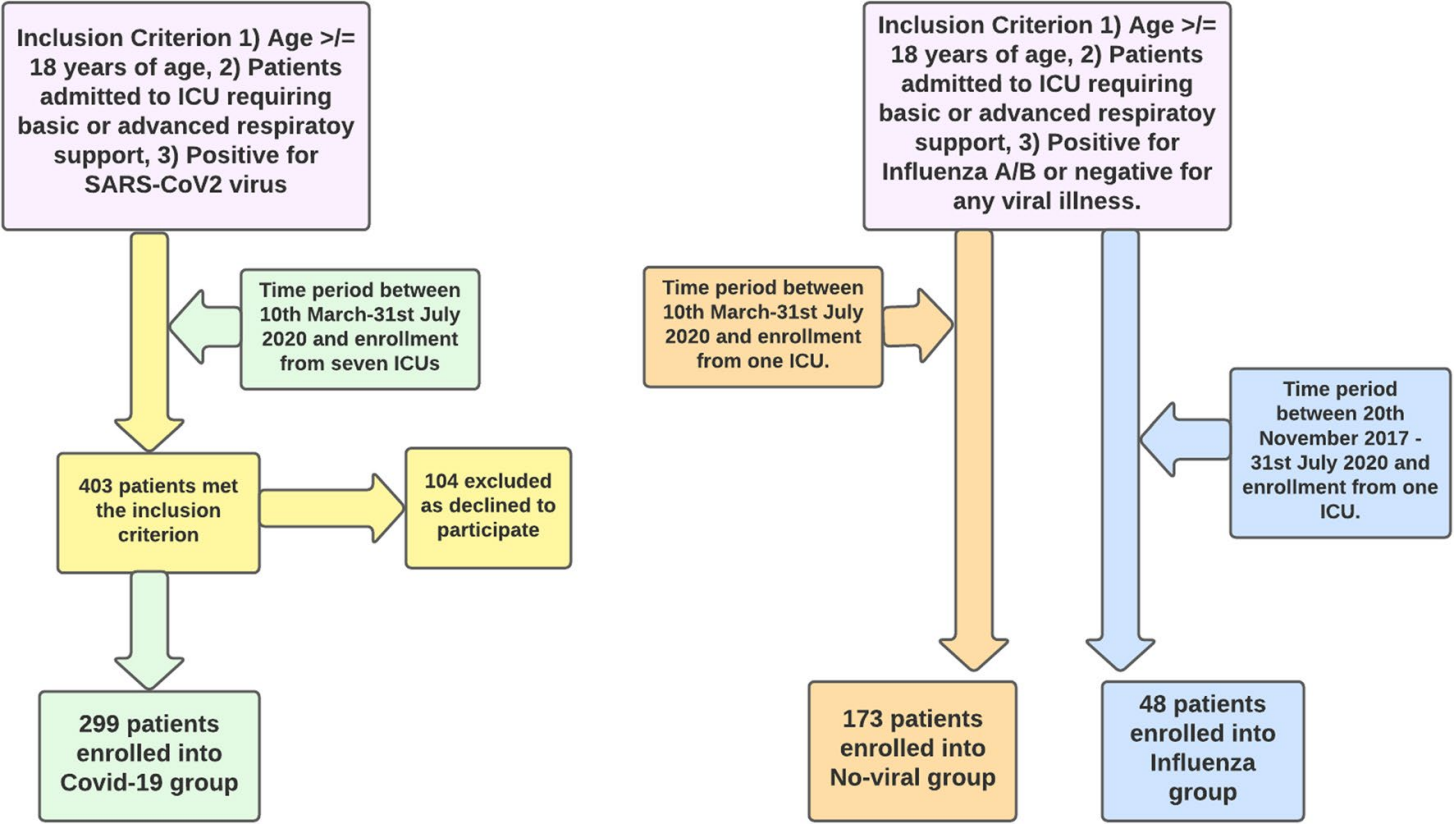
Flow chart illustrating inclusion criterion and number of patients recruited into each arm

Consecutive patients with SARS-CoV-2 pneumonia admitted to seven participating ICUs out of twelve commissioned ICUs in Wales were included during the first wave of the pandemic from 10 March until 31 July 2020. SARS-CoV-2 viral infection was confirmed by polymerase chain reaction (PCR) testing of either throat swab, nasopharyngeal swab or respiratory secretions. Non-matched data for patients with no viral pneumonia and Influenza A or B patients needing ICU admission for basic or advanced respiratory support were taken from one large ICU located at Cardiff (Fig. 1). We included patients who required ICU admission with Influenza A or B, starting from year 2020 and going back retrospectively in time up until year 2017. The timeline for non-matched Influenza A or B patient cohort was from 20 November 2017 to 31 July 2020, a period of three consecutive winter flu peaks. Lastly, the no viral pneumonia cohort was consecutive patients admitted to ICU during the first COVID-19 wave for medical or surgical indications, specifically without positive PCRs for SARS-CoV-2 and Influenza A or B and requiring basic or advanced respiratory support (Additional file 1: Fig. S4).

Prior agreement for the study was approved by each individual university teaching hospital as a service evaluation project. The study involved use of routine clinical data with waiving of the requirement of patient informed consent. This study followed the ‘Strengthening the Reporting of Observational Studies in Epidemiology (STROBE)’ statement guidelines for observational cohort studies [15].

### Study populations and data collection

Data from seven ICUs were pooled together on a single electronic data acquisition system. Recorded patient data were extracted from individual ICUs electronic ward watcher database. Data extracted included demographics (age, gender, body mass index {BMI}, ethnicity), clinical frailty score (CFS), admission APACHE-II (Acute Physiology and Chronic Health Evaluation-II) score, admission PaO_2_/FiO_2_ ratio, type and days of organ support required during ICU admission, primary reason for ICU admission, percentage of patients requiring basic and advanced respiratory support, percentage of patients requiring basic and advanced cardiac support, percentage of patients requiring Renal Replacement Therapy (RRT), comorbidities (percentage of patient with severe respiratory and cardiac comorbidity), median time elapsed from hospital to ICU admission, median duration of ICU stay, ICU mortality and 28-day ICU mortality. The follow-up of patients was completed until 30 September 2020.

The microbiology data of individual patients were extracted from an electronic data repository (data store) within Public Health Wales as per existing Information Governance agreements between the organisation and each individual ICUs in Wales. Microbiology data were linked with patient demographic details to provide a matching dataset. The microbiology data included type of organism grown from each culture sample type, number of culture sample types sent during ICU stay (blood or respiratory samples including sputum/non-directed bronchoalveolar lavage {NBAL}/bronchoalveolar lavage {BAL}) and antibiotic susceptibility testing of organism identified from sample, date and time of both samples sent from ICU and their individual results.

### Microbiology methods and definitions

All samples were processed as per the Wales Microbiology Standard Operating Procedure based on the UK Standards for Microbiology Investigations B57 Investigation of bronchoalveolar lavage, sputum and associated specimens. Microbiological susceptibility testing was performed by standard testing protocols utilising EUCAST (European Committee on Antimicrobial Susceptibility Testing) methodology. This was completed at National Health Service (NHS) laboratories associated with the participating ICUs. Isolated organisms were excluded if considered to represent commensals or contaminants. This includes blood cultures isolating common skin organisms (*Coagulase negative Staphylococci*, *Corynebacterium spp.* and *Cutibacterium spp.*) and respiratory samples isolating *Candida spp.* or non-pathogenic organisms commonly found within the oral flora (oral *Streptococci, Neisseria spp., Aggregatibacter spp., Rothia spp.*) or common colonisers in the context of ICU patients (*Coagulase negative Staphylococci and Corynebacterium spp.*).

### Outcome

The primary outcome of our study was the rate of early and late bloodstream (BSI) and respiratory tract (RTI) infection on a per patient and per sample basis in all three cohorts (COVID-19, Influenza A or B and no viral pneumonia). Secondary outcomes were ascertaining antibiotic sensitivity, and any correlation between ICU mortality with clinical or demographic variables during ICU admission.

### Statistical methods

Descriptive analyses are presented for COVID-19, Influenza A or B and no viral pneumonia groups. Continuous data are presented as median (IQR) as data were found to be non-normally distributed (Table 1). We considered only the first ICU admission event for any patient who was subsequently re-admitted to ICU in the specified time frame (repeat ICU admissions: COVID-19 n = 14, no viral pneumonia n = 5, Influenza n = 2).

**Table 1:** Demographics, admission parameters, level of organ support, ICU days and ICU mortality for COVID-19, no viral pneumonia and Influenza groups

	COVID-19	‘No viral’	Influenza
Number of patients	299	173	48
Age	57 (16)	53 (25)	58 (24)
Female (%)	33.8	34.7	54.2
BMI	28.7 (8.5)	25.8 (6.3)	24.5 (5.3)
Ethnicity: White–British (%)	83.3	90.8	91.7
*Frailty (%)*			
Group A	93.3	87.3	66.7
Group B	5.4	7.5	22.9
Group C	1.0	2.9	6.2
Group D	0.3	0.6	4.2
Severe cardiovascular comorbidity (%)	1.0	0.6	0.0
Severe respiratory comorbidity (%)	1.3	0.6	4.2
*Primary reason for admission (%)*			
Medical	100	50.3	100
Neurological	0	45.1	0
Surgical	0	4.6	0
Admission Apache-II	14.0 (7.0)	14.0 (10.0)	16.5 (9.0)
Admission P/F ratio	133.0 (83.0)	242.5 (163.1)	133.9 (92.3)
Time from hospital admission to ICU admission (days)	0.0 (5.2)	0.0 (7.2)	0.0 (3.5)
Admitted to ICU within 24 h of hospital admission (%)	69.2	79.8	72.9
Admitted to ICU within 48 h of hospital admission (%)	79.9	83.8	81.3
Requiring basic respiratory support (FM, NIMV) (%)	51.8	13.3	75.0
Days of basic respiratory support (FM, NIMV)	1.0 (3.0)	0.0 (0.0)	1.0 (2.2)
Requiring advanced respiratory support (IMV) (%)	75.9	100.0	60.4
Days of advanced respiratory support (IMV)	10.0 (19.5)	5.0 (11.0)	3.0 (11.5)
Requiring basic cardiovascular support (%)	95.3	98.8	95.8
Days of basic cardiovascular support	12.0 (17.0)	7.0 (10.0)	5.5 (9.2)
Requiring advanced cardiovascular support (%)	20.4	21.4	14.6
Days of advanced cardiovascular support	0.0 (0.0)	0.0 (0.0)	0.0 (0.0)
Requiring RRT (%)	30.4	19.1	20.8
Days of RRT	0.0 (2.0)	0.0 (0.0)	0.0 (0.0)
Length of ICU stay (median days)	13 (15.8)	7.1 (14.4)	7 (16.0)
ICU 28-day mortality (%)	30.8	26.0	25.0
ICU total mortality (%)	34.8	28.3	27.1

All continuous data are given as median (IQR), all categorical data as proportion (%)

BMI: Body mass index; frailty: grouped by level of prehospital dependency, A is most independence, D is lowest independence; admission APACHE-II: APACHE-II scores on admission to ICU; P/F ratio: PaO2/FiO2 ratio on admission to ICU; RRT: renal replacement therapy; APACHE-II: Acute Physiology and Chronic Health Evaluation-II; ICU: intensive care unit, FM: face mask, NIMV: non-invasive mechanical ventilation, IMV: invasive mechanical ventilation

Microbiology results from the same time period were uniquely matched to patient admissions via unique NHS number and date of birth. Blood cultures and respiratory secretions (including sputum, BAL and NBAL) were included. Samples taken more than 5 days prior to hospital admission date were excluded from analysis. De-duplication was performed on the microbiology results with each patient allowed to isolate a specific organism type once in each sample type (respiratory or blood culture). Any repeat positive results of the same organism in the same sample type in any one individual patient were removed. Microbiology results were divided into early (positive sample results within 48 h of hospital admission date) and late infection (positive sample results more than 48 h after hospital admission date). Logistic regressions were performed for each group (COVID-19, Influenza A or B and no viral pneumonia) with predictor variables of age, BMI, admission P/F ratio, admission APACHE-II score and gender. The outcome variable was no microbiological infection vs. microbiological infection (either early and/or late) (Additional file 1: Table 9). Assumption checks were met. All observations were individual, and predictor variables were not correlated with each other with Pearson’s R < 0.3 for all variables, thus meeting assumptions of no multicollinearity. Of note, the Influenza group was borderline with regard to assumption checks with 9.6 patients per predictor (48 patients, 5 predictor variables). All the models created for each group are explained in detail in Additional file (Additional file 1: Table 9).

All analyses were performed in R (v3.6.1) with the following packages utilised: tidyverse (v1.3.0), lubridate (v1.7.9.2), ggplot2 (v3.3.3), dbplyr (v1.0.5), plyr (v1.8.6) and scales (v1.1.1).

## Results

### Patient characteristics

In total, 520 patients were included in the comparative analysis (20 November 2017–31 July 2020) (Fig. 1). A total of 299 patients met the inclusion criterion and were positive for SARS-CoV-2 virus from seven participating ICUs. A total of 173 and 48 patients met the inclusion criterion for no viral pneumonia and Influenza A or B, respectively, from one participating ICU (Additional file 1: Fig. S4). Demographic data, median days from hospital to ICU admission, admission APACHE-II and P/F ratio, percentage of organ support needed during ICU stay, median duration of ICU stay and 28-day mortality data are detailed in Table 1. Patients in the COVID-19 group had higher BMI, lower admission P/F ratio as compared to the Influenza cohort which had the highest median admission APACHE-II score. Median duration of advanced respiratory support requirements was ten days in COVID-19 cohort as compared to five and three days for no viral pneumonia and Influenza A or B cohort, respectively.

### Microbiological results

#### Results by per culture sample sent

In total, 771 blood cultures and 602 respiratory cultures samples were sent from the COVID-19 patient group for microbiological analysis. Respiratory samples from the COVID-19 patient group consisted of 291 sputum, 43 bronchial lavage and 268 non-directed bronchial lavage samples (Additional file 1: Table S8). Total numbers and significant culture positivity rate of respiratory and blood culture specimens sent within and after 48 h of hospital admission in each patient group are listed in Table 2a, b and Additional file 1: Table 7. The proportion of late respiratory samples (collected after more than 48 h in hospital) isolating a pathogen was higher in COVID-19 patient group compared to the no viral pneumonia group (49.3% vs 38.8%, Chi-squared p = 9.16 × 10^–3^) but lower in the COVID-19 patient group compared to the influenza group (49.3% vs 64.9%, Chi-squared p = 0.03) (Table 2b). The proportion of positive early respiratory samples and both early and late blood cultures was similar between the 3 groups (Table 2a, b).

**Table 2:** (a, b) Percentage positivity (%) of early and late infection in blood and respiratory culture samples

(a)						
Group	Blood cultures					
	Early infection (< 48 h)			Late infection (> 48 h)		
	Total number of blood culture specimens	Number of cultures with significant organisms	Percent positivity (%)	Total number of blood culture specimens	Number of cultures with significant organisms	Percent positivity (%)
COVID-19	155	5	3.2	616	56	9.1
‘No viral’	57	5	8.8	224	25	11.2
Influenza	73	4	5.5	111	11	9.9

(b)						
Group	Respiratory samples					
	Early infection (< 48 h)			Late infection (> 48 h)		
	Total number of respiratory samples	Number of cultures with significant organisms	Percent positivity (%)	Total number of respiratory samples	Number of cultures with significant organisms	Percent positivity (%)
COVID-19	93	25	26.9	509	251	49.3
‘No viral’	76	26	34.2	219	85	38.8
Influenza	30	9	30.0	57	37	64.9

#### Results by per patient basis

Proportion of patients with early infection was lower in the COVID-19 group (8.7%) compared to the no viral pneumonia cohort (14.5%, Chi-squared p = 0.05) and the influenza cohort (25.0% Chi-squared p = 7.9 × 10^–4^). The proportion of patients isolating a co-pathogen by the end of their ICU stay was higher in the COVID-19 cohort (47.2%) compared to the no viral pneumonia cohort (37.6%, Chi-squared p = 0.04) but not compared to the Influenza group (50.0%, Chi-squared p = 0.71), although the Influenza group was relatively small. Late respiratory co-infections were higher within the COVID-19 cohort (35.5%) than the no viral pneumonia cohort (24.3%, Chi-squared p = 0.01) and the influenza cohort (31.3%, Chi-squared p = 0.57). Late blood stream infection was also higher in the COVID-19 patient cohort (13.4%) than the no viral pneumonia cohort (5.8%, Chi-squared p = 7.0 × 10^–3^) but not the influenza cohort (14.6%, Chi-squared p = 0.82) (Table 3).

**Table 3:** (n—total number of patients, %—proportion out of total for three groups, namely COVID-19, Influenza and no viral)

		COVID-19		‘No viral’		Influenza	
		n	%	n	%	N	%
Patients		299	100	173	100	48	100
Early Infection	Patients w/ positive blood cultures	5	1.7	3	1.7	4	8.3
	Patients w/ significant respiratory culture	22	7.4	22	12.7	8	16.7
	Patients w/ any significant culture	26	8.7	25	14.5	12	25.0
Late Infection	Patients w/ positive blood cultures	40	13.4	10	5.8	7	14.6
	Patients w/ significant respiratory culture	106	35.5	42	24.3	15	31.3
	Patients w/ any significant culture	123	41.1	47	27.2	16	33.3
Infection at any time	Patients w/ positive blood cultures	45	15.1	13	7.5	10	20.8
	Patients w/ significant respiratory culture	121	40.5	60	34.7	20	41.7
	Patients w/ any significant culture	141	47.2	65	37.6	24	50.0

**Fig. 2:**
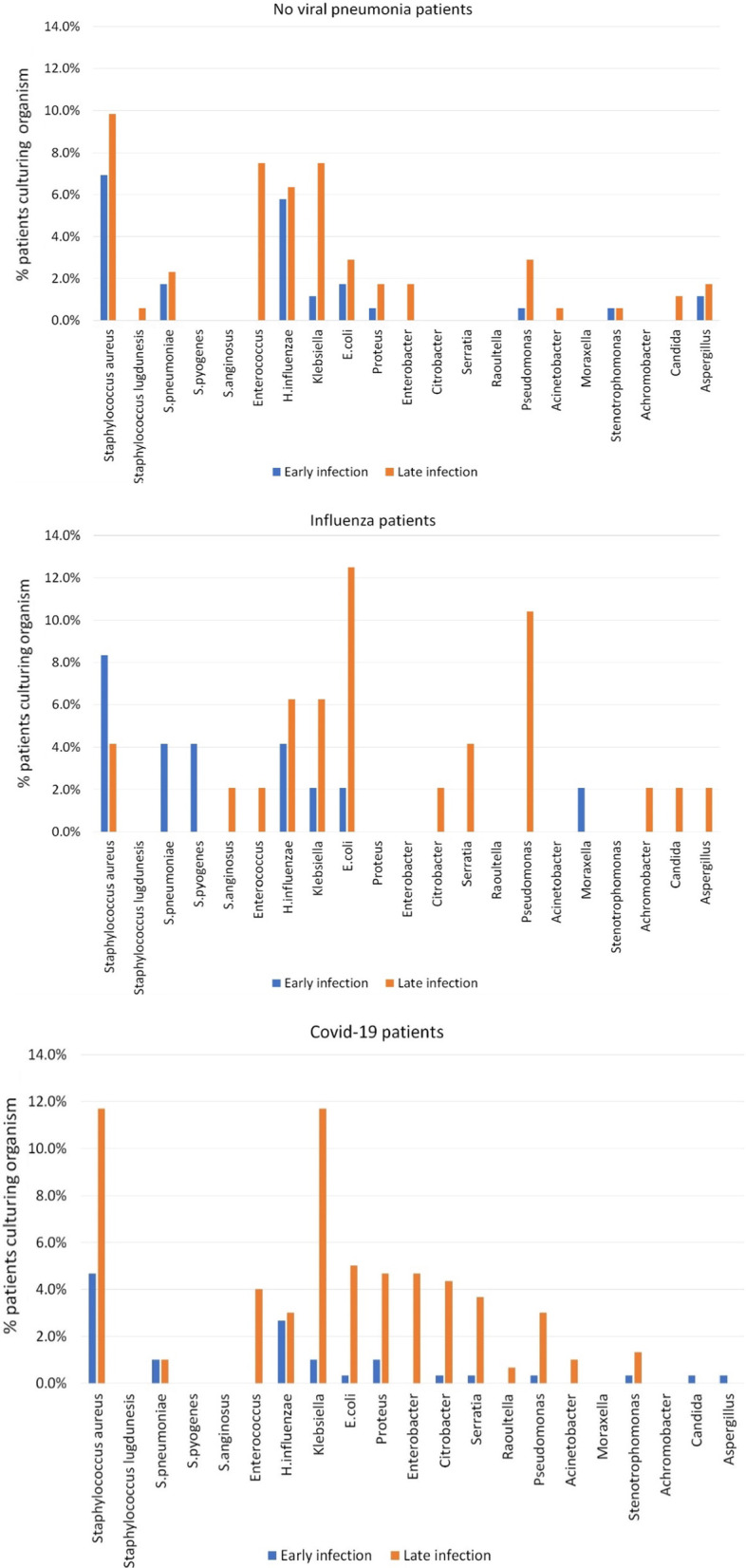
Bar diagrams for COVID-19, Influenza and no viral pneumonia cohorts demonstrating cultured organism in early and late infection, % of individual patients culturing organism on Y-axis

**Fig. 3:**
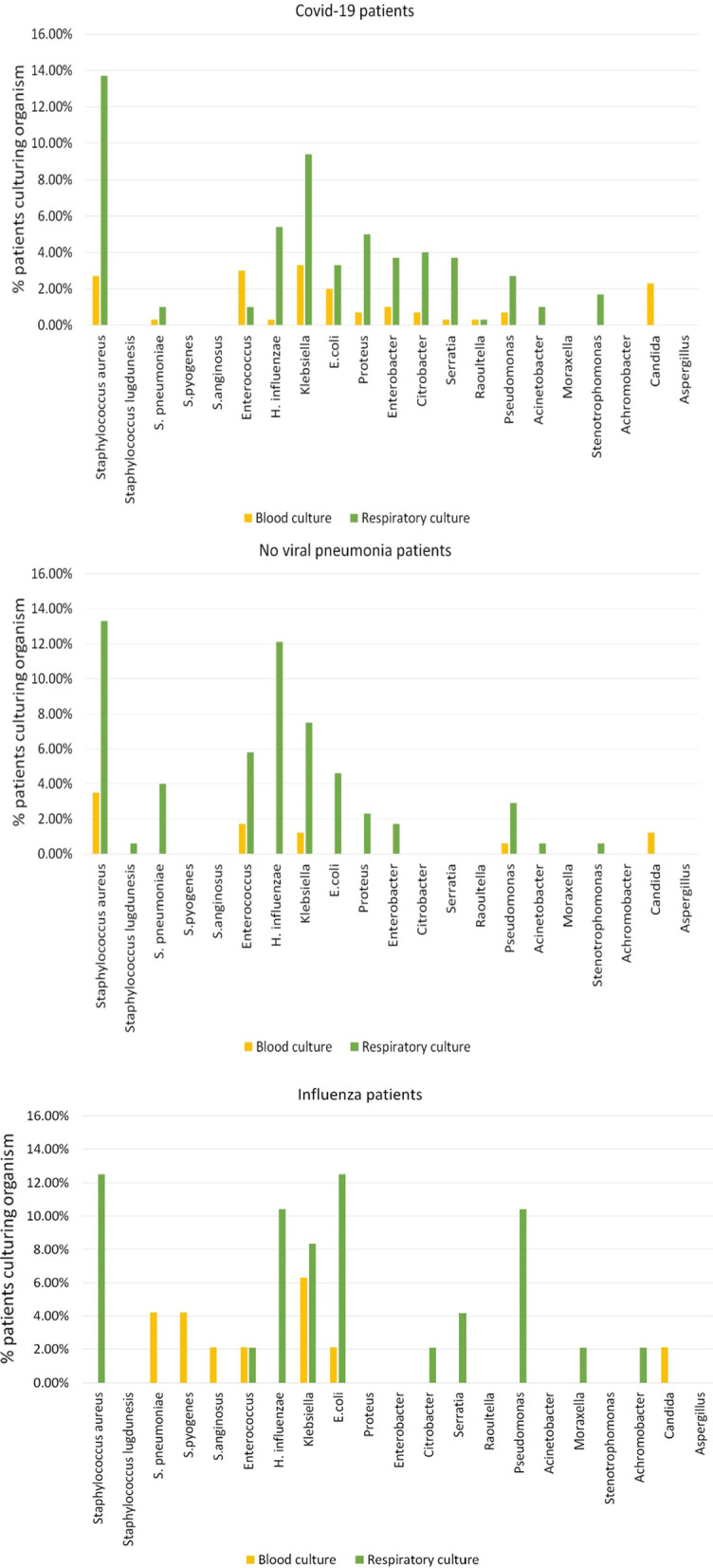
Bar diagrams for COVID-19, Influenza and no viral pneumonia cohorts demonstrating cultured organisms from blood or respiratory tract specimens, % of individual patients culturing organism on Y-axis

Figure 2 demonstrates the specific co-pathogens isolated within 48 h (early infection) and after 48 h of admission (late infection) as a percentage of patients in each group. Figure 3 demonstrates the co-pathogens isolated by microbiological sample type as percentage of patients in each group. Absolute numbers of each organism are listed above the bars in each figure. *Staphylococcus aureus* was the commonest co-pathogen isolated within 48 h of admission in each patient group (Additional file 1: Table S4). For late infections more than 48 h into admission, in the COVID-19 group *Staphylococcus aureus* and *Klebsiella spp.* were most common with 35 (11.7%) patients each (Fig. 2) (Additional file 1: Table S4). In the no viral pneumonia group, late infection with *Staphylococcus aureus* was most common occurring in 17 (9.8%) patients, followed by *Klebsiella spp.* and *Enterococci spp*. with 13 (7.5%) patients each (Fig. 2) (Additional file 1: Table S4). In the Influenza group, the majority of late infections were caused by Gram-negative organisms with 6 (12.5%) patients isolating *E. coli* and 5 (10.4%) patients isolating *Pseudomonas aeruginosa* (Fig. 2) (Additional file 1: Table S4). Within the COVID-19 group, 7 (2.3%) patients isolated *Aspergillus* from respiratory samples with similar proportions of patients isolating this organism in the other groups. Seven (2.3%) COVID-19 patients had a *Candida* bloodstream infection (Fig. 3). *Candida* bloodstream infection was identified in 1 (2.1%) Influenza patient and 2 (1.2%) no viral pneumonia patients (Additional file 1: Table S5).

#### Antimicrobial susceptibility testing results

Antimicrobial susceptibility testing results for the isolated co-pathogens for selected agents are found in Additional file 1: Table 6. Three out of 49 (6.1%) *S. aureus* isolates were MRSA (methicillin-resistant *S. aureus*) in the COVID-19 group. One out of 6 (16.7%) S. aureus isolates was MRSA in the Influenza cohort, whilst there were none in the no viral pneumonia group. All MRSA isolates were from respiratory samples. A single patient isolated a penicillin-resistant Streptococcus pneumoniae in a respiratory sample from the COVID-19 group. This resistance was not seen in the other groups. In the COVID-19 group, 3 out of 116 (2.6%) first isolates of *Enterobacterales* (2 *Enterobacter spp*. and 1 *Serratia liquefaciens*) demonstrated third-generation cephalosporin resistance. One of the resistant *Enterobacter spp.* isolates was from a blood culture. Third-generation cephalosporin resistance in *Enterobacterales* was demonstrated in 2 out of 30 (6.7%) isolates in the no viral pneumonia group and 4 out of 17 (23.5%) isolates in the Influenza group. No meropenem-resistant *Enterobacterales* were isolated in any of the groups. Two out of 10 (20.0%) *Pseudomonas spp*. first isolates in the COVID-19 group demonstrated meropenem resistance. In the no viral pneumonia group, 2 out of 6 (33.3%) *Pseudomonas spp.* isolates demonstrated meropenem resistance and in the Influenza group 2 out of 5 (40.0%) *Pseudomonas spp*. isolates demonstrated this resistance (Additional file 1: Table S6a–f). Incidence of multi-drug resistance (MDR) organism isolated from blood and the respiratory specimens for all three groups are described in Additional file 1: Table 10. There was no organism with extensively drug resistance (XDR) identified [34].

#### Multivariate analyses

With regard to the logistic regressions, none of the models for any of the groups proved significant in predicting microbiological infection vs. no microbiological infection. For COVID-19 and no viral pneumonia group, the overall models containing sex, age, APACHE-II, BMI and P/F ratio did not significantly predict the presence of our outcome variable of an added microbiological infection (COVID-19: X2(246) = 6.712, p > 0.05, Nagelkerke R2 = 0.035; and no viral pneumonia: X2(167) = 2.896, p > 0.05, Nagelkerke R2 = 0.023). Of the predictor factors in the COVID-19 and no viral pneumonia models, none were significant individual predictors of our outcome variable (all p > 0.05). For the Influenza group (n = 48), the model also did not significantly predict the presence of added microbiological infection vs. no infection (Influenza: X2(40) = 3.717, p > 0.05, Nagelkerke R2 = 0.105). Of the predictor variables, none were significant individual predictors of the outcome variable (all p > 0.05) (Additional file 1: Table S9).

## Discussion

We report the results of a largest comparative ICU cohort study on the rate of early and late BSI or RTI in patients admitted to ICUs in Wales with COVID-19 pneumonia requiring basic or advanced respiratory support. The study compares microbiological sampling results with non-matched ICU patient cohort admitted with Influenza A or B pneumonia or no viral pneumonia cohort, respectively.

In our study, we have clearly defined separation between timing (> 48 h vs < 48 h) of sample positivity from date of hospitalisation and site (bloodstream and respiratory tract) of sampling. 80% of the total COVID-19 cohort (n-299) were admitted to ICUs within 48 h of hospitalisation. Previous published studies and rapid reviews have quoted variable rates of bacterial and fungal co-infection in hospitalised patients with COVID-19 pneumonia (3.5–14.0%) without differentiating between source of isolated pathogen (bloodstream or respiratory) and timing of pathogen isolated from the time of hospitalisation [14, 17–22]. Only two of these studies have reported co-infection data from critically ill COVID-19 cohorts, and the rate of co-infection in these severely unwell ICU cohorts was between 8.1% and 14.0% [18, 19]. Early rates of co-infection within our COVID-19 cohort (8.7%) are similar to these previously published studies. Our study demonstrates a higher proportion of COVID-19 patients having evidence of co-infection by end of their ICU stay (47.2%). The majority of co-infections in the COVID-19 cohort were late respiratory tract infections. Our study is more in keeping with studies containing only COVID-19 patients on ICU that have reported rates of co-infection of 28% at the time of admission to ICU [23] and 50.5% of patients ventilated for more than week developing respiratory co-infection [24]. The proportion of patients with BSI in the COVID-19 cohort (15.1%) was similar to recently published international EPIC-III data, with a lower proportion seen in the comparator no viral pneumonia cohort (7.5%) [30].

The higher rate of late co-infection observed in COVID-19 cohort may be partially explained by patient characteristics with higher median days of advanced respiratory support requirement and lower admission P/F ratio with moderate-to-severe AHRF on ICU admission. The data were also collected from first wave of COVID-19 pandemic. During this period, there was evidence of higher usage of empiric antibiotic cover for a novel respiratory viral disease which might have contributed to isolating Gram-negative organism later in the course of ICU stay [27]. There was also the challenge of acute pressure of caring for a higher number of acutely sick patients in a short interval with higher patient to nursing ratios (> 1:1) and need to provide care whilst wearing personal protective equipment (PPE). Using adjunctive measures to improve oxygenation like prone ventilation might have contributed to higher burden of late RTI [28].

Gram-positive cocci, mostly MSSA (methicillin-sensitive *Staphylococcus aureus*), were the predominant isolated pathogen within 48 h of hospitalisation. Gram-negative bacilli mainly from Enterobacterales group were predominantly isolated after 48 h in all three cohorts. This is in keeping with organisms identified in other COVID-19 ICU patients [23, 24]. Low rates of antimicrobial resistance were observed in all 3 cohorts. The rate of acquiring MRSA in our COVID-19 cohort was less as compared to previously reported rates in patients with SARS pneumonia [16]. Similar proportions of patients in each group cultured *Aspergillus* in respiratory samples. Diagnosing pulmonary aspergillosis in patients on respiratory support requires correlation with fungal biomarker tests and imaging sinuses and lung parenchyma. Pulmonary aspergillosis is frequently diagnosed in the absence of *Aspergillus* culture and is a recognised complication of both COVID-19 and severe Influenza A or B [25, 26].

Our study provides evidence to support the recommended choice of antibiotics for hospitalised COVID-19 patients with suspected co-infection in NICE guidelines [31, 33]. It also supports the locally developed antibiotic regime of flucloxacillin, amoxicillin and clarithromycin as empiric therapy to COVID-19 patients with suspected secondary bacterial pneumonia from the community that was used in the hospitals associated with ICUs in the study. The higher rates of late co-infection observed in COVID-19 cohort highlight a need to continue to monitor these patients, whilst they are on ICUs for signs of co-infection and promptly start appropriate antibiotics when co-infection is suspected.

Strengths of this study include its multicentre design creating a large cohort of ICU patients admitted with COVID-19 pneumonia with data for early and late BSI and RTI co-infection and the availability of comparator data from ICU patients with Influenza A or B pneumonia and no viral pneumonia. Detailed microbiological data including antimicrobial susceptibility are available for culture results taken from blood and respiratory secretions from these patients with these being reported in relation to time from hospital admission.

Limitations of this study include retrospective study design with inherent bias towards microbiological sampling rate. Data from the comparator influenza and no viral pneumonia patient cohorts are non-propensity matched and limited to a single centre. This has resulted in these cohorts being smaller, and particularly influenza cohort and comparisons between it and the COVID-19 cohort should be interpreted with caution. The heterogeneous nature of the no viral pneumonia cohort should also be noted in interpreting comparisons with it. Antimicrobial prescribing data were unavailable for all cohorts during the entire ICU and hospital stay, and it is one of the biggest limitations of the study. The antibiotic treatment for all patients was chosen either empirically or based upon culture sample and its antibiotic sensitivity results. In spite of the above antibiotic prescribing approach, overall prevalence rate of co-infection was more or less similar to the previously published International Multicentric Study [30]. Prior exposure to antimicrobial therapy has the potential to select for organisms resistant to the given antimicrobial therapy. Part of the rationale of the early co-infection cut-off of 48 h from hospital admission was to minimise impact of any prior antibiotic exposure on identified organisms. De-duplication steps in our data collection methodology have the potential to conceal emerging antibiotic resistance in patients. The number of prone ventilated days has not been captured, and this has been demonstrated to increase rates of respiratory tract infection elsewhere [28]. Rates of co-infection within our study are potential overestimates as isolating pathogens in respiratory samples from a ventilated patient does not necessarily imply infection. During the study inclusion period, data for steroid prescription have not been captured and patients might have received steroid as part of a research trial, but only 3/299 patients in the COVID cohort received steroid as part of treatment after the press release for dexamethasone arm of RECOVERY trial [32]. The COVID-19 cohort data were collected from the first wave of the pandemic. Since then, there have been significant developments in the treatment and outcomes of patients with COVID-19 and a large proportion of the general population is vaccinated against SARS-CoV-2 and these changes need to be considered in applying the findings of this study to current COVID-19 patients.

## Conclusion

The proportion of ICU patient with co-infection was higher with COVID-19 pneumonia as compared to those without viral pneumonia. This mostly was due to higher rates of late (more than 48 h from hospital admission) respiratory tract infection, but also higher rates of late blood stream infection were observed. To enable identification of any potential modifiable risk factor, a large prospective study will be required in the future.

## Supplementary Information

**Additional file 1**.** Table 4**. Demonstrating number of individual pathogens isolated, separated by early vs late.** Table 5**. Demonstrating number of individual pathogens isolated, separated by source of culture i.e, blood or respiratory.** Figure 3**. demonstrating number of patients recruited from individual hospitals.** Table 6**. Demonstrating antibiotic susceptibility of different pathogen in all three groups.** Table 7**. Demonstrating cumulative rate of BSI and RTI by per sample basis.** Table 8**. Demonstrating types of respiratory samples.** Table 9**. Demonstrating baseline characteristics for patients who are classified into two groups of with co-infection and without co-infection.** Table 10**. Demonstrating resistance pattern for all three groups.

## Data Availability

All data needed to evaluate the conclusions in this article are present and tabulated in the main text or Additional file 1: Appendix. This article is the result of an original retrospective cohort. For individual de-identified raw data that underlie the results reported in this article, please contact the corresponding author. The de-identified patient data could be made available, and it will need individual hospital ethical committee permission prior to sharing with editors.
