# Supplementary material for: Comparative incidence of early and late bloodstream and respiratory tract co-infection in patients admitted to ICU with COVID-19 pneumonia versus Influenza A or B pneumonia versus no viral pneumonia: wales multicentre ICU cohort study
Source: Crit Care. 2022 Jun 2;26:158. doi: 10.1186/s13054-022-04026-9 (PMC9160852; doi:10.1186/s13054-022-04026-9)
Supplement: Supplementary file 1 — Additional file 1. Table 4. Demonstrating number of individual pathogens isolated, separated by early vs late. Table 5. Demonstrating number of individual pathogens isolated, separated by source of culture i.e, blood or respiratory. Figure 3. demonstrating number of patients recruited from individual hospitals. Table 6. Demonstrating antibiotic susceptibility of different pathogen in all three groups. Table 7. Demonstrating cumulative rate of BSI and RTI by per sample basis. Table 8. Demonstrating types of respiratory samples. Table 9. Demonstrating baseline characteristics for patients who are classified into two groups of with co-infection and without co-infection. Table 10. Demonstrating resistance pattern for all three groups. [file 13054_2022_4026_MOESM1_ESM.docx]

**Comparative incidence of early and late bloodstream and respiratory tract co-infection in patients admitted to ICU with COVID-19 pneumonia versus Influenza A or B pneumonia versus no viral illness: Wales multi-centre ICU cohort study**

**Supplementary reference: -**

1. **Table-4 demonstrating number of individual pathogens isolated, separated by early vs late.**
2. **Table-5 demonstrating number of individual pathogens isolated, separated by source of culture i.e, blood or respiratory.**
3. **Figure -3 demonstrating number of patients recruited from individual hospitals.**
4. **Table 6 demonstrating antibiotic susceptibility of different pathogen in all three groups**
5. **Table 7: - demonstrating cumulative rate of BSI and RTI by per sample basis**
6. **Table 8: - demonstrating types of respiratory samples**
7. **Table 9: - demonstrating baseline characteristics for patients who are classified into two groups of with co-infection and without co-infection.**
8. **Table 10: - demonstrating resistance pattern for all three groups.**

| **Organism** | **Early Infection** | | | | | | **Late Infection** | | | | | |
| --- | --- | --- | --- | --- | --- | --- | --- | --- | --- | --- | --- | --- |
|  | **Covid-19** | | **No-Viral** | | **Influenza** | | **Covid-19** | | **No-Viral** | | **Influenza** | |
|  | n | % | n | % | n | % | n | % | n | % | n | % |
| *Staphylococcus aureus* | 14 | 4.7 | 12 | 6.9 | 4 | 8.3 | 35 | 11.7 | 17 | 9.8 | 2 | 4.2 |
| *Staphylococcus lugdunesis* | 0 | 0.0 | 0 | 0.0 | 0 | 0.0 | 0 | 0.0 | 1 | 0.6 | 0 | 0.0 |
| *Streptococcus pneumoniae* | 3 | 1.0 | 3 | 1.7 | 2 | 4.2 | 3 | 1.0 | 4 | 2.3 | 0 | 0.0 |
| *Streptococcus pyogenes* | 0 | 0.0 | 0 | 0.0 | 2 | 4.2 | 0 | 0.0 | 0 | 0.0 | 0 | 0.0 |
| *Streptococcus anginosus* | 0 | 0.0 | 0 | 0.0 | 0 | 0.0 | 0 | 0.0 | 0 | 0.0 | 1 | 2.1 |
| *Enterococcus spp* | 0 | 0.0 | 0 | 0.0 | 0 | 0.0 | 12 | 4.0 | 13 | 7.5 | 1 | 2.1 |
| *Haemophilus influenzae* | 8 | 2.7 | 10 | 5.8 | 2 | 4.2 | 9 | 3.0 | 11 | 6.4 | 3 | 6.3 |
| *Klebsiella spp* | 3 | 1.0 | 2 | 1.2 | 1 | 2.1 | 35 | 11.7 | 13 | 7.5 | 3 | 6.3 |
| *Escherichia coli* | 1 | 0.3 | 3 | 1.7 | 1 | 2.1 | 15 | 5.0 | 5 | 2.9 | 6 | 12.5 |
| *Proteus spp* | 3 | 1.0 | 1 | 0.6 | 0 | 0.0 | 14 | 4.7 | 3 | 1.7 | 0 | 0.0 |
| *Enterobacter cloacae* | 0 | 0.0 | 0 | 0.0 | 0 | 0.0 | 14 | 4.7 | 3 | 1.7 | 0 | 0.0 |
| *Citrobacter spp* | 1 | 0.3 | 0 | 0.0 | 0 | 0.0 | 13 | 4.3 | 0 | 0.0 | 1 | 2.1 |
| *Serratia spp* | 1 | 0.3 | 0 | 0.0 | 0 | 0.0 | 11 | 3.7 | 0 | 0.0 | 2 | 4.2 |
| *Raoultella spp* | 0 | 0.0 | 0 | 0.0 | 0 | 0.0 | 2 | 0.7 | 0 | 0.0 | 0 | 0.0 |
| *Pseudomonas aeuroginosa* | 1 | 0.3 | 1 | 0.6 | 0 | 0.0 | 9 | 3.0 | 5 | 2.9 | 5 | 10.4 |
| *Acinetobacter baumannii* | 0 | 0.0 | 0 | 0.0 | 0 | 0.0 | 3 | 1.0 | 1 | 0.6 | 0 | 0.0 |
| *Moraxella catarrhalis* | 0 | 0.0 | 0 | 0.0 | 1 | 2.1 | 0 | 0.0 | 0 | 0.0 | 0 | 0.0 |
| *Stenotrophomonas maltophila* | 1 | 0.3 | 1 | 0.6 | 0 | 0.0 | 4 | 1.3 | 1 | 0.6 | 0 | 0.0 |
| *Achromobacter sp* | 0 | 0.0 | 0 | 0.0 | 0 | 0.0 | 0 | 0.0 | 0 | 0.0 | 1 | 2.1 |
| *Candida spp* | 1 | 0.3 | 0 | 0.0 | 0 | 0.0 | 6 | 2.0 | 2 | 1.2 | 1 | 2.1 |
| *Aspergillus spp* | 1 | 0.3 | 2 | 1.2 | 0 | 0.0 | 6 | 2.0 | 3 | 1.7 | 1 | 2.1 |

**Table 4: -** Where *n* indicates the number of patients isolating the named organism, and *%* indicates the percentage of patients isolating the named organisms within the specified group.

| **Organism** | **Blood Culture** | | | | | | **Respiratory Culture** | | | | | |
| --- | --- | --- | --- | --- | --- | --- | --- | --- | --- | --- | --- | --- |
|  | **Covid-19** | | **No-Viral** | | **Influenza** | | **Covid-19** | | **No-Viral** | | **Influenza** | |
|  | n | % | n | % | n | % | n | % | n | % | n | % |
| *Staphylococcus aureus* | 8 | 2.7 | 6 | 3.5 | 0 | 0.0 | 41 | 13.7 | 23 | 13.3 | 6 | 12.5 |
| *Staphylococcus lugdunesis* | 0 | 0.0 | 0 | 0.0 | 0 | 0.0 | 0 | 0.0 | 1 | 0.6 | 0 | 0.0 |
| *Streptococcus pneumoniae* | 1 | 0.3 | 0 | 0.0 | 2 | 4.2 | 3 | 1.0 | 7 | 4.0 | 0 | 0.0 |
| *Streptococcus pyogenes* | 0 | 0.0 | 0 | 0.0 | 2 | 4.2 | 0 | 0.0 | 0 | 0.0 | 0 | 0.0 |
| *Streptococcus anginosus* | 0 | 0.0 | 0 | 0.0 | 1 | 2.1 | 0 | 0.0 | 0 | 0.0 | 0 | 0.0 |
| *Enterococcus spp* | 9 | 3.0 | 3 | 1.7 | 1 | 2.1 | 3 | 1.0 | 10 | 5.8 | 1 | 2.1 |
| *Haemophilus influenzae* | 1 | 0.3 | 0 | 0.0 | 0 | 0.0 | 16 | 5.4 | 21 | 12.1 | 5 | 10.4 |
| *Klebsiella spp* | 10 | 3.3 | 2 | 1.2 | 3 | 6.3 | 28 | 9.4 | 13 | 7.5 | 4 | 8.3 |
| *Escherichia coli* | 6 | 2.0 | 0 | 0.0 | 1 | 2.1 | 10 | 3.3 | 8 | 4.6 | 6 | 12.5 |
| *Proteus spp* | 2 | 0.7 | 0 | 0.0 | 0 | 0.0 | 15 | 5.0 | 4 | 2.3 | 0 | 0.0 |
| *Enterobacter cloacae* | 3 | 1.0 | 0 | 0.0 | 0 | 0.0 | 11 | 3.7 | 3 | 1.7 | 0 | 0.0 |
| *Citrobacter spp* | 2 | 0.7 | 0 | 0.0 | 0 | 0.0 | 12 | 4.0 | 0 | 0.0 | 1 | 2.1 |
| *Serratia spp* | 1 | 0.3 | 0 | 0.0 | 0 | 0.0 | 11 | 3.7 | 0 | 0.0 | 2 | 4.2 |
| *Raoultella spp* | 1 | 0.3 | 0 | 0.0 | 0 | 0.0 | 1 | 0.3 | 0 | 0.0 | 0 | 0.0 |
| *Pseudomonas aeruginosa* | 2 | 0.7 | 1 | 0.6 | 0 | 0.0 | 8 | 2.7 | 5 | 2.9 | 5 | 10.4 |
| *Acinetobacter baumannii* | 0 | 0.0 | 0 | 0.0 | 0 | 0.0 | 3 | 1.0 | 1 | 0.6 | 0 | 0.0 |
| *Moraxella catarrhalis* | 0 | 0.0 | 0 | 0.0 | 0 | 0.0 | 0 | 0.0 | 0 | 0.0 | 1 | 2.1 |
| *Stenotrophomonas maltophila* | 0 | 0.0 | 0 | 0.0 | 0 | 0.0 | 5 | 1.7 | 1 | 0.6 | 0 | 0.0 |
| *Achromobacter sp* | 0 | 0.0 | 0 | 0.0 | 0 | 0.0 | 0 | 0.0 | 0 | 0.0 | 1 | 2.1 |
| *Candida spp* | 7 | 2.3 | 2 | 1.2 | 1 | 2.1 | - | - | - | - | - | - |
| *Aspergillus spp* | 0 | 0.0 | 0 | 0.0 | 0 | 0.0 | 7 | 2.3 | 5 | 2.9 | 2 | 4.2 |

**Table -5: -** Where *n* indicates the number of patients isolating the named organism, and *%* indicates the percentage of patients isolating the named organisms within the specified group.

**Figure 4**: - n- Number of patients recruited from individual acute care hospitals

**
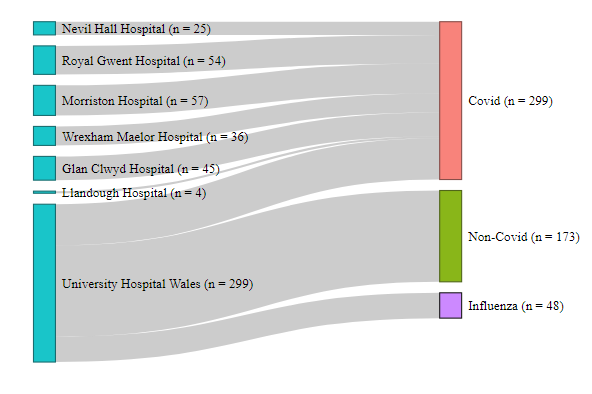
**

**Table 6: -** Antibiotic susceptibility of different pathogens in all three groups (Covid-19, Influenza and no-viral).

Table 6a: - Covid-19 blood culture isolate susceptibilities: -

| **Antibiotic** | **S. aureus** | | | **S. pneumoniae** | | | **H. influenzae** | | | **P. aeuroginosa** | | | **Enterobacterales** | | |
| --- | --- | --- | --- | --- | --- | --- | --- | --- | --- | --- | --- | --- | --- | --- | --- |
|  | **S (n)** | **Isolates tested (n)** | **S (%)** | **S (n)** | **Isolates tested (n)** | **S (%)** | **S (n)** | **Isolates tested (n)** | **S (%)** | **S (n)** | **Isolates tested (n)** | **S (%)** | **S (n)** | **Isolates tested (n)** | **S (%)** |
| **Doxycycline** | **8** | **8** | **100** | **1** | **1** | **100** | **1** | **1** | **100** |  |  |  |  |  |  |
| **Amoxicillin** |  |  |  | **1** | **1** | **100** | **1** | **1** | **100** |  |  |  | **6** | **25** | **24** |
| **Co-amoxiclav** | **8** | **8** | **100** | **1** | **1** | **100** | **1** | **1** | **100** |  |  |  | **18** | **25** | **72** |
| **Levofloxacin** | **8** | **8** | **100** | **1** | **1** | **100** | **1** | **1** | **100** |  |  |  |  |  |  |
| **Clarithromycin** | **6** | **8** | **75** | **1** | **1** | **100** |  |  |  |  |  |  |  |  |  |
| **Cefuroxime** | **8** | **8** | **100** | **1** | **1** | **100** | **1** | **1** | **100** |  |  |  | **24** | **25** | **96** |
| **Flucloxacillin** | **8** | **8** | **100** |  |  |  |  |  |  |  |  |  |  |  |  |
| **Co-trimoxazole** | **8** | **8** | **100** | **1** | **1** | **100** | **1** | **1** | **100** |  |  |  | **23** | **25** | **92** |
| **Ciprofloxacin** |  |  |  |  |  |  |  |  |  | **2** | **2** | **100** | **24** | **25** | **96** |
| **Gentamicin** |  |  |  |  |  |  |  |  |  | **2** | **2** | **100** | **25** | **25** | **100** |
| **Ceftazidime** |  |  |  |  |  |  |  |  |  | **2** | **2** | **100** |  |  |  |
| **Piperacillin/Tazobactam** |  |  |  |  |  |  |  |  |  | **2** | **2** | **100** | **22** | **25** | **88** |
| **Ertapenem** |  |  |  |  |  |  |  |  |  |  |  |  | **24** | **25** | **96** |
| **Meropenem** |  |  |  |  |  |  |  |  |  | **1** | **2** | **50** | **25** | **25** | **100** |

Table 6b: -Covid-19 respiratory culture isolate susceptibilities: -

| **Antibiotic** | **S. aureus** | | | **S. pneumoniae** | | | **H. influenzae** | | | **P. aeuroginosa** | | | **Enterobacterales** | | |
| --- | --- | --- | --- | --- | --- | --- | --- | --- | --- | --- | --- | --- | --- | --- | --- |
|  | **S (n)** | **Isolates tested (n)** | **S (%)** | **S (n)** | **Isolates tested (n)** | **S (%)** | **S (n)** | **Isolates tested (n)** | **S (%)** | **S (n)** | **Isolates tested (n)** | **S (%)** | **S (n)** | **Isolates tested (n)** | **S (%)** |
| **Doxycycline** | **41** | **41** | **100** | **2** | **3** | **67** | **16** | **16** | **100** |  |  |  |  |  |  |
| **Amoxicillin** |  |  |  | **2** | **3** | **67** | **7** | **16** | **44** |  |  |  | **14** | **91** | **15** |
| **Co-Amoxiclav** | **38** | **41** | **93** | **2** | **3** | **67** | **11** | **16** | **69** |  |  |  | **50** | **91** | **55** |
| **Levofloxacin** | **21** | **25** | **84** | **3** | **3** | **100** | **14** | **16** | **88** |  |  |  |  |  |  |
| **Clarithromycin** | **28** | **41** | **68** | **2** | **3** | **67** |  |  |  |  |  |  |  |  |  |
| **Cefuroxime** | **38** | **41** | **93** | **3** | **3** | **100** | **16** | **16** | **100** |  |  |  | **89** | **91** | **98** |
| **Flucloxacillin** | **38** | **41** | **93** |  |  |  |  |  |  |  |  |  |  |  |  |
| **Co-Trimoxazole** | **25** | **25** | **100** | **2** | **2** | **100** | **11** | **16** | **69** |  |  |  | **80** | **91** | **88** |
| **Ciprofloxacin** |  |  |  |  |  |  |  |  |  | **8** | **8** | **100** | **84** | **91** | **92** |
| **Gentamicin** |  |  |  |  |  |  |  |  |  | **8** | **8** | **100** | **88** | **91** | **97** |
| **Ceftazidime** |  |  |  |  |  |  |  |  |  | **8** | **8** | **100** |  |  |  |
| **Piperacillin/Tazobactam** |  |  |  |  |  |  |  |  |  | **7** | **8** | **88** | **84** | **91** | **92** |
| **Ertapenem** |  |  |  |  |  |  |  |  |  |  |  |  | **89** | **90** | **99** |
| **Meropenem** |  |  |  |  |  |  |  |  |  | **7** | **8** | **88** | **91** | **91** | **100** |

Table 6c: -No viral blood culture isolate susceptibilities: -

| **Antibiotic** | **S. aureus** | | | **P. aeuroginosa** | | | **Enterobacterales** | | |
| --- | --- | --- | --- | --- | --- | --- | --- | --- | --- |
|  | **S (n)** | **Isolates tested (n)** | **S (%)** | **S (n)** | **Isolates tested (n)** | **S (%)** | **S (n)** | **Isolates tested (n)** | **S (%)** |
| **Doxycycline** | **6** | **6** | **100** |  |  |  |  |  |  |
| **Amoxicillin** |  |  |  |  |  |  | **0** | **2** | **0** |
| **Co-Amoxiclav** | **6** | **6** | **100** |  |  |  | **2** | **2** | **100** |
| **Levofloxacin** | **5** | **6** | **83** |  |  |  |  |  |  |
| **Clarithromycin** | **4** | **6** | **67** |  |  |  |  |  |  |
| **Cefuroxime** | **6** | **6** | **100** |  |  |  | **2** | **2** | **100** |
| **Flucloxacillin** | **6** | **6** | **100** |  |  |  |  |  |  |
| **Co-Trimoxazole** | **6** | **6** | **100** |  |  |  | **2** | **2** | **100** |
| **Ciprofloxacin** |  |  |  | **1** | **1** | **100** | **2** | **2** | **100** |
| **Gentamicin** |  |  |  | **1** | **1** | **100** | **2** | **2** | **100** |
| **Ceftazidime** |  |  |  | **1** | **1** | **100** |  |  |  |
| **Piperacillin/Tazobactam** |  |  |  | **1** | **1** | **100** | **2** | **2** | **100** |
| **Ertapenem** |  |  |  |  |  |  | **2** | **2** | **100** |
| **Meropenem** |  |  |  | **1** | **1** | **100** |  |  |  |

Table 6d: -No viral respiratory culture isolate susceptibilities

| **Antibiotic** | **S. aureus** | | | **S. pneumoniae** | | | **H. influenzae** | | | **P. aeruginosa** | | | **Enterobacterlaes** | | |
| --- | --- | --- | --- | --- | --- | --- | --- | --- | --- | --- | --- | --- | --- | --- | --- |
|  | **S (n)** | **Isolates tested (n)** | **S (%)** | **S (n)** | **Isolates tested (n)** | **S (%)** | **S (n)** | **Isolates tested (n)** | **S (%)** | **S (n)** | **Isolates tested (n)** | **S (%)** | **S (n)** | **Isolates tested (n)** | **S (%)** |
| **Doxycycline** | **23** | **23** | **100** | **7** | **7** | **100** | **21** | **21** | **100** |  |  |  |  |  |  |
| **Amoxicillin** |  |  |  | **7** | **7** | **100** | **10** | **21** | **48** |  |  |  | **8** | **28** | **29** |
| **Co-Amoxiclav** | **23** | **23** | **100** |  |  |  | **13** | **21** | **62** |  |  |  | **17** | **28** | **41** |
| **Levofloxacin** | **19** | **21** | **90** | **7** | **7** | **100** | **19** | **21** | **90** |  |  |  |  |  |  |
| **Clarithromycin** | **20** | **23** | **87** | **7** | **7** | **100** |  |  |  |  |  |  |  |  |  |
| **Cefuroxime** | **23** | **23** | **100** |  |  |  | **21** | **21** | **100** |  |  |  | **26** | **28** | **93** |
| **Flucloxacillin** | **23** | **23** | **100** |  |  |  |  |  |  |  |  |  |  |  |  |
| **Co-Trimoxazole** | **22** | **22** | **100** | **4** | **4** | **100** | **19** | **21** | **90** |  |  |  | **26** | **28** | **93** |
| **Ciprofloxacin** |  |  |  |  |  |  |  |  |  | **5** | **5** | **100** | **27** | **28** | **96** |
| **Gentamicin** |  |  |  |  |  |  |  |  |  |  |  |  | **26** | **28** | **93** |
| **Ceftazidime** |  |  |  |  |  |  |  |  |  | **4** | **5** | **80** |  |  |  |
| **Piperacillin/Tazobactam** |  |  |  |  |  |  |  |  |  | **4** | **5** | **80** | **25** | **28** | **89** |
| **Ertapenem** |  |  |  |  |  |  |  |  |  |  |  |  | **28** | **28** | **100** |
| **Meropenem** |  |  |  |  |  |  |  |  |  | **3** | **5** | **60** | **28** | **28** | **100** |

Table 6e: -Influenza blood culture isolate susceptibilities

| **Antibiotic** | **Streptococcus pneumoniae** | | | **Enterobacterales** | | |
| --- | --- | --- | --- | --- | --- | --- |
|  | **S (n)** | **Isolates tested (n)** | **S (%)** | **S (n)** | **Isolates tested (n)** | **S (%)** |
| **Doxycycline** | **2** | **2** | **100** |  |  |  |
| **Amoxicillin** | **2** | **2** | **100** | **0** | **4** | **0** |
| **Co-Amoxiclav** | **2** | **2** | **-** | **3** | **4** | **75** |
| **Levofloxacin** | **2** | **2** | **100** |  |  |  |
| **Clarithromycin** | **2** | **2** | **100** |  |  |  |
| **Cefuroxime** | **2** | **2** | **100** | **2** | **4** | **50** |
| **Flucloxacillin** |  |  |  |  |  |  |
| **Co-Trimoxazole** | **2** | **2** | **100** | **2** | **4** | **50** |
| **Ciprofloxacin** |  |  |  | **2** | **4** | **50** |
| **Gentamicin** |  |  |  | **4** | **4** | **100** |
| **Ceftazidime** |  |  |  |  |  |  |
| **Piperacillin/Tazobactam** |  |  |  | **3** | **4** | **75** |
| **Ertapenem** |  |  |  | **4** | **4** | **100** |
| **Meropenem** |  |  |  | **4** | **4** | **100** |

Table 6f: -Influenza respiratory culture isolate susceptibilities

| **Antibiotic** | **S. aureus** | | | **H. influenzae** | | | **P. aeuroginosa** | | | **Enterobacterales** | | |
| --- | --- | --- | --- | --- | --- | --- | --- | --- | --- | --- | --- | --- |
|  | **S (n)** | **Isolates tested (n)** | **S (%)** | **S (n)** | **Isolates tested (n)** | **S (%)** | **S (n)** | **Isolates tested (n)** | **S (%)** | **S (n)** | **Isolates tested (n)** | **S (%)** |
| **Doxycycline** | **6** | **6** | **100** | **5** | **5** | **100** |  |  |  |  |  |  |
| **Amoxicillin** |  |  |  | **3** | **5** | **60** |  |  |  | **5** | **13** | **38** |
| **Co-Amoxiclav** | **5** | **6** | **83** | **5** | **5** | **100** |  |  |  | **10** | **13** | **77** |
| **Levofloxacin** | **6** | **6** | **100** | **5** | **5** | **100** |  |  |  |  |  |  |
| **Clarithromycin** | **5** | **6** | **83** |  |  |  |  |  |  |  |  |  |
| **Cefuroxime** | **5** | **6** | **83** | **5** | **5** | **100** |  |  |  | **11** | **13** | **85** |
| **Flucloxacillin** | **5** | **6** | **83** |  |  |  |  |  |  |  |  |  |
| **Co-Trimoxazole** | **6** | **6** | **100** | **2** | **5** | **40** |  |  |  | **11** | **13** | **85** |
| **Ciprofloxacin** |  |  |  |  |  |  | **5** | **5** | **100** | **12** | **13** | **92** |
| **Gentamicin** |  |  |  |  |  |  |  |  |  | **13** | **13** | **100** |
| **Ceftazidime** |  |  |  |  |  |  | **4** | **5** | **80** |  |  |  |
| **Piperacillin/Tazobactam** |  |  |  |  |  |  | **4** | **5** | **80** | **13** | **13** | **100** |
| **Ertapenem** |  |  |  |  |  |  |  |  |  | **13** | **13** | **100** |
| **Meropenem** |  |  |  |  |  |  | **3** | **5** | **60** | **13** | **13** | **100** |

**Table 7: –** Cumulative rate of BSI and RTI by per sample basis.

| **Group** | **Blood Cultures** | | | **Respiratory Samples** | | |
| --- | --- | --- | --- | --- | --- | --- |
|  | **Total number of blood culture specimens** | **Number of significant organisms isolated** | **Percent positivity (%)** | **Total number of respiratory specimens** | **Number of significant organisms isolated** | **Percent positivity (%)** |
| **Covid** | **771** | **61** | **7.91** | **602** | **276** | **45.85** |
| **No viral** | **281** | **30** | **10.68** | **295** | **111** | **37.63** |
| **Influenza** | **184** | **15** | **8.15** | **87** | **46** | **52.87** |

**Table 8: - Types of respiratory sample processed for each patient group**

| **Group** | **Sputum** | **Bronchial lavage** | **Non-directed bronchial lavage** | **Total** |
| --- | --- | --- | --- | --- |
| **Covid** | **291** | **43** | **268** | **602** |
| **No viral** | **55** | **46** | **194** | **295** |
| **Influenza** | **34** | **15** | **38** | **87** |

Table 9: - Baseline characteristics of patients for all three cohorts and individual cohort classification into two groups of coinfection and No coinfection.

|  | **COVID** | | | **No viral** | | | **Influenza** | |
| --- | --- | --- | --- | --- | --- | --- | --- | --- |
|  | **Co-infection** | | **No co-infection** | **Co-infection** | **No co-infection** | | **Co-infection** | **No co-infection** |
| **Number of patients** | **141** | | **158** | **65** | **108** | | **24** | **24** |
| Age | 56 (13) | | 58 (18) | 53 (20) | 54 (27) | | 64 (17) | 55 (22) |
| Female (%) | 35.9 | | 31.3 | 32.3 | 36.1 | | 54.2 | 54.2 |
| BMI | 29.3 (8.7) | | 28.4 (7.3) | 25.5 (6.3) | 25.9 (6.3) | | 24.8 (4.4) | 23.8 (7.0) |
| Ethnicity: White – British (%) | 81.6 | | 85.4 | 87.7 | 92.6 | | 87.5 | 95.8 |
| Frailty (%): |  | |  |  |  | |  |  |
| Group A | 95.7 | | 91.1 | 84.1 | 91.6 | | 75.0 | 58.3 |
| Group B | 3.5 | | 7.0 | 9.5 | 6.5 | | 20.8 | 25.0 |
| Group C | 0.7 | | 1.3 | 4.8 | 1.9 | | 4.2 | 8.3 |
| Group D | 0.0 | | 0.6 | 1.6 | 0.0 | | 0.0 | 8.3 |
| Severe cardiovascular comorbidity (%) | 0.7 | | 1.3 | 0.0 | 0.9 | | 0.0 | 0.0 |
| Severe respiratory comorbidity (%) | 0.0 | | 2.5 | 0.0 | 0.9 | | 0.0 | 8.3 |
|  |  | |  |  |  | |  |  |
| Primary reason for admission (%): | |  | | | |  | | |
| Medical | 100 | | 100 | 50.7 | 50.0 | | 100 | 100 |
| Neurological | 0 | | 0 | 40.0 | 41.7 | | 0 | 0 |
| Surgical | 0 | | 0 | 9.3 | 8.3 | | 0 | 0 |
| Admission Apache II | 13.0 (5.5) | | 14.0 (7.5) | 13.0 (7.0) | 14.0 (11.0) | | 18.5 (8.0) | 15.0 (5.5) |
| Admission P/F ratio | 125.4 (76.6) | | 144.7 (83.1) | 248.8 (184.8) | 239.0 (175.7) | | 118.2 (107.1) | 130.3 (93.5) |
| Time from hospital admission to ICU admission (days) | 0.0 (2.0) | | 1.0 (2.0) | 0.0 (1.0) | 0.0 (1.0) | | 0.0 (2.0) | 0.0 (1.3) |
| Admitted to ICU within 24 hours of hospital admission (%) | 70.9 | | 67.5 | 80.0 | 78.7 | | 70.8 | 75.0 |
| Admitted to ICU within 48 hours of hospital admission (%) | 80.1 | | 77.7 | 83.1 | 83.3 | | 83.3 | 79.2 |
|  |  | |  |  |  | |  |  |
| Requiring basic respiratory support (%) | 47.5 | | 56.1 | 23.1 | 7.4 | | 54.2 | 91.7 |
| Days of basic respiratory support | 0.0 (3.0) | | 1.0 (3.0) | 0.0 (0.0) | 0.0 (0.0) | | 1.0 (1.3) | 2.5 (3.0) |
| Requiring advanced respiratory support (%) | 97.2 | | 56.7 | 100.0 | 100.0 | | 83.3 | 37.5 |
| Days of advanced respiratory support | 18.0 (18.0) | | 2.0 (10.0) | 12.0 (17.0) | 4.0 (5.3) | | 10.0 (22.0) | 0.0 (5.3) |
| Requiring basic cardiovascular support (%) | 97.2 | | 93.0 | 100.0 | 98.1 | | 95.8 | 95.8 |
| Days of basic cardiovascular support | 20.0 (17.0) | | 6.0 (9.0) | 12.0 (15.0) | 5.0 (7.0) | | 11.5 (20.8) | 4.0 (3.3) |
| Requiring advanced cardiovascular support (%) | 25.5 | | 15.9 | 36.9 | 12.0 | | 20.8 | 12.5 |
| Days of advanced cardiovascular support | 0.0 (1.0) | | 0.0 (0.0) | 0.0 (2.0) | 0.0 (0.0) | | 0.0 (0.0) | 0.0 (0.0) |
| Requiring RRT (%) | 36.2 | | 25.5 | 33.8 | 10.2 | | 29.2 | 12.5 |
| Days of RRT | 0.0 (6.0) | | 0.0 (1.0) | 0.0 (5.0) | 0.0 (0.0) | | 0.0 (1.3) | 0.0 (0.0) |
|  |  | |  |  |  | |  |  |
| Length of ICU stay (median days) | 21.0 (17.0) | | 6.0 (10.0) | 14.3 (18.0) | 4.6 (7.7) | | 13.1 (19.1) | 4.7 (4.0) |
| ICU 28- day mortality (%) | 28.4 | | 33.1 | 24.6 | 26.9 | | 20.8 | 29.2 |
| ICU total mortality (%) | 36.2 | | 33.8 | 29.2 | 27.8 | | 25.0 | 29.2 |

Table 10: Proportion of microbiological isolates meeting the proposed joint European Centre for Disease Prevention and Control (ECDC) and the Centers for Disease Control and Prevention (CDC) interim standard definitions for multidrug-resistant (MDR) bacteria

Table 10a: Proportion of MDR Blood culture isolates

| **Organism** | **Covid-19** | | | **No-Viral** | | | **Influenza** | | |
| --- | --- | --- | --- | --- | --- | --- | --- | --- | --- |
|  | Isolates | MDR (n) | MDR (%) | Isolates | MDR (n) | MDR (%) | Isolates | MDR (n) | MDR (%) |
| *Staphylococcus aureus* | 8 | 0 | 0 | 6 | 0 | 0 | 0 | - | - |
| *Enterococcus spp* | 9 | 0 | 0 | 3 | 1 | 33.3 | 1 | 0 | 0 |
| *Enterobacterales* | 25 | 1 | 4 | 2 | 0 | 0 | 4 | 1 | 25 |
| *Pseudomonas aeuroginosa* | 2 | 0 | 0 | 1 | 0 | 0 | 0 | - | - |
| *Acinetobacter baumannii* | 0 | - | - | 0 | - | - | 0 | - | - |

Table 10b: Proportion of MDR Respiratory culture isolates

| **Organism** | **Covid-19** | | | **No-Viral** | | | **Influenza** | | |
| --- | --- | --- | --- | --- | --- | --- | --- | --- | --- |
|  | Isolates | MDR (n) | MDR (%) | Isolates | MDR (n) | MDR (%) | Isolates | MDR (n) | MDR (%) |
| *Staphylococcus aureus* | 41 | 3 | 7.3 | 23 | 0 | 0 | 6 | 1 | 16.7 |
| *Enterococcus spp* | 3 | 0 | 0 | 10 | 2 | 20 | 1 | 0 | 0 |
| *Enterobacterales* | 91 | 4 | 4.4 | 28 | 3 | 10.7 | 13 | 0 | 0 |
| *Pseudomonas aeuroginosa* | 8 | 0 | 0 | 5 | 0 | 0 | 5 | 1 | 20 |
| *Acinetobacter baumannii* | 3 | 0 | 0 | 1 | 0 | 0 | 0 | - | - |
